# The Impact of Hemispheric Activity Priming on Choking Under Pressure in Badminton Tasks: A Study of Three Fundamental Skills

**DOI:** 10.5114/jhk/173023

**Published:** 2023-10-27

**Authors:** Wei Wang, Melanie J. Gregg, Hairui Liu

**Affiliations:** 1 College of Physical Education, Hubei Normal University, Huangshi, China.; 2 Department of Kinesiology & Applied Health, University of Winnipeg, Winnipeg, Canada.; 3 Department of Education, Health & Behavior Studies, University of North Dakota, Grand Forks, USA.

**Keywords:** motor skills, hand squeezing, badminton fundamental movement skills

## Abstract

Choking under pressure occurs when an individual experiences a decrease in performance despite their efforts to perform well. The self-focus approach suggests that pressure increases conscious attention on the performance process, disrupting the automatic or overlearned nature of execution. Hemispheric asymmetries in the brain and skilled performance indicate that left-hemispheric activity decreases, while right-hemispheric activity enhances. Previous studies have attempted to prevent choking by inhibiting the left hemisphere or enhancing the right hemisphere's activity. This study examined whether increased hemispheric activity priming can extenuate motor skill failure under pressure in badminton tasks. The study involved 32 right-handed college students who completed five conditions in pressure-free blocks versus choking under-pressure blocks with priming intervention. Results showed a significant improvement in motor learning from pre- to post-tests, but participants still choked under pressure during skill execution. Furthermore, the priming strategy (hand squeezing) did not alleviate the pressure to benefit performance. The study provides evidence of performance decrements under pressure conditions, and the priming strategy did not alleviate choking.

## Introduction

During the FIFA World Cup, 2022, the pressure was on as second ranked Belgium was required to best twelfth ranked Croatia to move on in the tournament, a Belgium striker was “faced with a wide-open net multiple times in the second half. And missed each time.” (McKeone, 2022). When an outcome is important, and the pressure mounting, athletes can experience anxiety, a narrowing of attentional focus, and a resulting catastrophic performance (Mesagno and Beckmann, 2017). Choking under pressure refers to performance decrements under pressure conditions despite an individual striving to perform well (Gröpel and Mesagno, 2019). Choking will only occur when the individual perceives the outcome as important, but choking is not inevitable. While there is no perfect solution for preventing choking in sport, there are some techniques that have been used successfully to decrease the likelihood that choking will occur. The key is to prevent choking from occurring in the first place.

Gröpel and Mesagno (2019) identified three effective intervention methods to improve performance under pressure: distraction (e.g., pre-performance routines), self-focus (e.g., left hand squeeze), and acclimatization (i.e., adapt to coping with increasing performance pressure). The present study used a self-focus intervention method, priming, giving the athletes a task-irrelevant dual task to prevent them from focusing on each step of a well learned motor task.

Responding to stimuli in our environment, such as taking a shot in a World Cup soccer tournament, can be primed to help lessen experiences of anxiety and ultimately lead to better skill execution and performance. Self-focus theory of choking posits that pressure to perform increases conscious attention on the process of performance and disrupts the automatic or overlearned nature of its execution (Gröpel and Mesagno, 2019; Masters and Maxwell, 2008). One of these self-focus theories is Reinvestment Theory which posits that the benefit of conscious attention varies depending on the stage of learning (Masters and Maxwell, 2008); novices tend to benefit from conscious attention whereas later in the learning process the reinvestment of conscious attention results in interference with the performance of motor tasks (Beckmann et al., 2021). In their work with Electroencephalography (EEG), researchers (Gallicchio et al., 2016) described high level athletes who experienced choking under pressure and had more interaction between the left temporal and frontal regions of the brain compared to athletes who did not experience choking, suggesting greater reinvestment of conscious attention. This increased brain activation suggests that during the choking process well learned motor skills have shifted from being unconscious, automatic movements, to conscious and it is this conscious processing that disrupts skilled performance. Cross-Villasana and colleagues (2015) used EEG to support their hypothesis that rather than a contralateral increase in activation, the self-focus technique caused an overall reduction in cortical excitability and resulted in a relaxation effect, allowing for automatic movements to occur.

According to hemispheric asymmetries in the brain and skilled performance, evidence (e.g., Beckmann et al., 2021; Hoskens et al., 2020) suggests that skilled performance reduces left-hemispheric activity and enhances right-hemispheric activity. Previous research reported the possibilities for preventing the choking effect under pressure conditions by inhibiting the activity of the left hemisphere or enhancing the activity of the right hemisphere (e.g., Beckmann et al., 2021; Hoskens et al., 2020). Beckmann et al. (2021) also attempted to use priming to facilitate stimulus processing induced by prior exposure to a related stimulus. Left-hand contractions have been identified as one of the most effective techniques for combating choking in sport (Beckmann et al., 2012; Gröpel and Mesagno, 2019) and this is the method used in the present study. Evidence for the efficacy of this technique was shown when right-handed junior tennis players who performed a left-hand dynamic handgrip prior to serving were able to maintain service accuracy when under pressure (Beckmann et al., 2021). In comparison, the group that performed a right-hand dynamic handgrip experienced a performance decrement.

Some researchers (Mesagno et al., 2019) suggest choking interventions must be adapted to the task demands of the sport. For example, badminton involves skills that must respond to an external stimulus (the shuttle) and being distracted or paying attention to the wrong cue can result in poor performance (Mesagno et al., 2019). Due to the unique characteristics of each sport, researchers such as Beckmann and colleagues (2021) recommend further research to examine the effect in a range of sports and specifically the sport of badminton due to the inherent pauses in the game, such as before serving, that may allow priming to occur. Beckmann et al. (2021) also recommend going beyond serving accuracy only to include additional measures of performance. Thus, the present study examined whether increased hemispheric activity priming would extenuate motor skill failure under choking in three distinct badminton tasks: a forehand clear, a serve, and a wall volley. The rationale behind the selection of these particular skills stems from two primary considerations: firstly, all three proficiencies hold paramount significance as foundational elements within the realm of badminton, serving as reliable indicators of an individual's practical competence and performance during both practice sessions and competitive gameplay (Liu et al., 2021); secondly, these skills have been recurrently employed and investigated in prior scholarly inquiries pertaining to the domain of badminton (Beckmann et al., 2012).

## Methods

### Participants

A cohort of 42 participants was enlisted from two existing badminton classes at a state university located in central China. These individuals were chosen based on specific criteria, including a minimum of two years of systematic badminton training and the selection of badminton as their primary sport during their physical education studies. The requirement of two years of badminton training served as a deliberate criterion to target students majoring in physical education who identified badminton as their principal sport for the purpose of this study. The study protocol (registered IRB #2021070021) was approved by the Institutional Review Board for Research Involving Human Subjects at the Hubei Normal University (protocol code: 2021070021; approval date: 15 August 2021) and all participants provided written informed consent prior to their participation in the study.

### Measures

*Handedness*. The Edinburgh Handedness Inventory (Oldfield, 1971) was utilized to evaluate the laterality of participants in the study. The inventory consists of ten items, including writing, drawing, throwing, using scissors, brushing teeth, using a knife (without a fork), using a spoon, sweeping with a broom, striking a match, and opening the lid of a box. Participants’ handedness was assessed by one of two methods: (1) self-report the preferred side to use for each of the items (self-rated method), or (2) the side used to be rated by an observer (direct observation method).

For each item, a “+” was marked in the column for the preferred side, and if the preference was strong to the point where the participant would only use that side unless forced, a “++” was marked. If there was no preference for either side, a “+” was marked on both columns. The final score, referred to as the Laterality Quotient, was calculated using the formula: Laterality Quotient = (R−L)/(R+L) X 100, where R and L represent the total number of “+” marks on the right and left columns, respectively. The Laterality Quotient was used to interpret handedness as follows: a value less than −40 indicated left-handedness, a value between −40 and +40 indicated ambidexterity, and a value greater than +40 indicated right-handedness.

**Figure 1:**
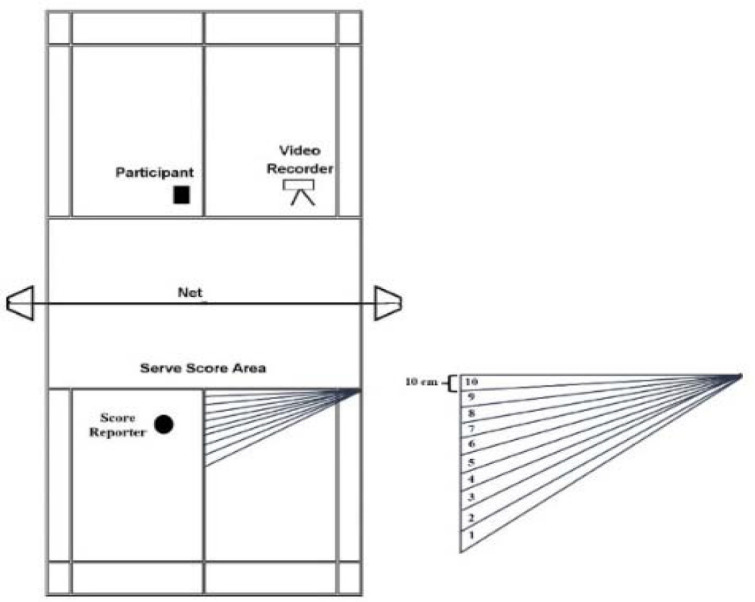
Serve Test.

*Skill tests*. For the Serve Test, participants executed ten serves from the right service court to the opponent's court in a game of badminton. The net was set according to international regulations, with a height of 1.55 m at the edges and 1.52 m in the center. This measure was designed for the present study in collaboration with a professional badminton coach; an optimal landing field was defined and marked as a target area on the opponent's left service court (as depicted in Figure 1). The performance of participants was evaluated by scoring the accuracy of each serve using a 10-point system. A serve that landed in the optimal field was awarded 10 points, while points decreased with the distance from the optimal field. Serves that landed outside the target or in the net were awarded 0 points. The maximum score in the serve test was 100 points.

**Figure 2:**
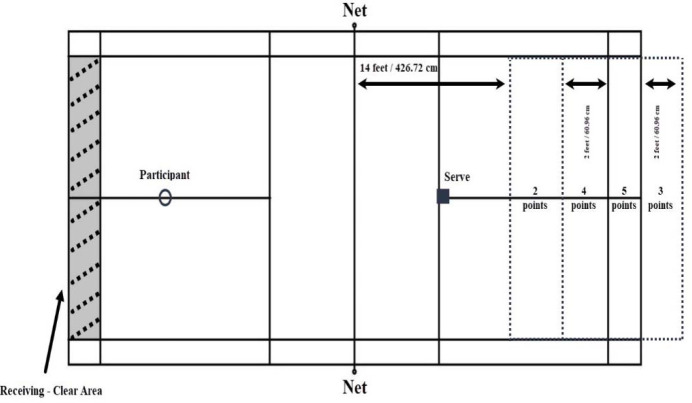
Forehand Clear Test.

The Forehand Clear Test (Liu et al., 2021) was used to assess the participants' ability to return a serve and hit the shuttle to the deepest part of the court (Figure 2). The court was divided into zones, and scores ranged from 0 to 5 based on the landing of the shuttlecock. Participants underwent ten trials, and the test was administered by a faculty member and expert badminton player for both the pre- and post-tests. The Forehand Clear Test was selected due to its reported reliability of 0.96 (odd/even correlation) and its ease of administration, as well as its relevance to the critical skill of successful badminton gameplay (Rink et al., 1996). The maximum score in the serve test was 50 points.

The Wall Volley Test was utilized to evaluate the participants' skill in terms of object control, a critical aspect of successful gameplay. The test, as described by Liu (2021), involved continuously hitting a shuttle to a wall for 30 s, scoring as many successful hits as possible. A successful hit was defined as one that landed above a line at net height from the floor and five feet from the wall. Participants were given two opportunities to complete the wall volley test, and their best score was recorded.

*State anxiety*. State anxiety was assessed using the Revised Competitive State Anxiety Inventory-2 (CSAI-2R; Cox et al., 2003). The somatic (7 items) and cognitive (5 items) subscales of the CSAI-2R were used in the analysis. Scores ranged from 10 to 40 for each subscale and items were rated on a 4-point Likert scale (1 = not at all, 2 = somewhat, 3 = moderately so, 4 = very much so). The validity of the CSAI-2R was established by Cox et al. (2003), with college-age athletes.

### Design and Procedures

Using a repeated within-subjects design, participants completed five conditions consisting of pressure-free blocks (pre- to post-measures) and choking under-pressure blocks (with no hand squeezing, left-hand squeezing, and right-hand squeezing) with a priming intervention. To minimize potential confounds related to fatigue or boredom on the measurements, only one series of repetitive hand contractions was performed for each hand, as previous research has indicated that a single series of contractions is sufficient for producing subsequent behavioral effects (Cross-Villasana et al., 2015).

In the pressure-free condition 1, participants first completed the Edinburgh Handedness Inventory (Oldfield, 1971). Subsequently, they engaged in an easy warm-up on the courts. The warm-up procedure commenced with a 10-min segment of running laps within the gymnasium, constituting a total of 10 laps. This initial phase was succeeded by an 8-min period focused on both static and dynamic stretches, targeting various joint areas. Following a 2-min hydration break, participants were organized into pairs and relocated to the badminton courts. Here, they engaged in 20 min of focused practice, emphasizing specific techniques including clears, serves, and volleys. The warm-up lasted approximately 40 min, incorporating general conditioning and badminton-specific drills and exercises. Participants were then individually called to the experimental court and were informed about the three skill tasks (a serve, a forehand clear, and a wall volley). The sequence of skill tests (a serve, a forehand clear, and a wall volley) was randomly determined by the coach using a number generator.

In the pressure-free condition 2, participants engaged in training sessions with their badminton coach over an eight-week period in the spring of 2022. Sessions occurred twice per week and lasted for 90 min each, resulting in a total of 1440 min of training and practice. The second test was administered, which mirrored the procedures of the first test, excluding the administration of the Edinburgh Handedness Inventory.

In the choking under-pressure conditions (conditions 3–5), participants were randomly assigned to two balanced teams (consisting of equal numbers of male and female participants) and instructed to compete against each other. The teams were informed the team with a higher score in each skill test would be rewarded with $100 (a total of $300 within three badminton skill tests). The teams performed the task on a rotating basis, with players from each team taking turns to complete the task. Verbal support from team members and discouragement of opponents was encouraged. Additionally, 100 spectators were introduced to the gym and divided into two groups to support and discourage each of the competing teams. To further enhance the pressure effect, a video camera was set up on the right side of the performer during the serve test, and participants were informed that their serving technique would be recorded and evaluated by their coach, requiring them to pay close attention to their technique (Liao and Masters, 2002).

In conditions 3–5, participants were informed of the aim to examine the effect of increased concentration on their skill execution accuracy, and hand-squeezing tasks were introduced as a simple method to promote it. Following the instructions, the competition commenced. The sequence of skill tests (a serve, a forehand clear, and a wall volley) and hand-squeezing (left-hand, right-hand, or no squeezing) were randomly determined by the coach using a number generator. There were two breaks of 10 min each between the three skill tests. For the hand-squeezing task, participants were instructed to squeeze a soft ball for approximately 30 s immediately prior to their turn. One research assistant helped to track for 30 s while instructing participants to squeeze the soft squeeze ball (size 58 mm * 44 mm, weight: 50 g) as hard as they can. To assess state anxiety, the CSAI-2R (Cox et al., 2003) was administered before and after the tasks.

### Statistical Analysis

Scatter plots were created to visually identify any outliers among the three dependent variables. Normality and homogeneity of variances were also assessed. A student's *t*-test was conducted to analyze the scores on the CSAI-2R in the choking under-pressure conditions (conditions 3–5). Three separate one-way analyses of variance (ANOVAs) were performed to determine any statistical differences between the effects of the different conditions on serve, forehand-clear, and wall volley scores. The significance of results obtained from the analyses was further evaluated with pairwise tests, using Bonferroni-adjusted alpha levels, and the effect sizes were determined with partial eta squared (η2).

Additionally, to evaluate the impact of the priming conditions (conditions 3–5: no hand squeezing, left-hand squeezing, right-hand squeezing), a Two One-Sided Test for Equivalence (TOST) was conducted, with the smaller standard deviation of the two distributions serving as the equivalence interval and an alpha level of 0.05 (Kirkpatrick et al., 2019). The uncorrected *p*-values were reported for each test. The TOST was used to test for statistical equivalence between the groups, with the aim of determining if any difference between the groups had a minimum effect size of 0.05.

## Results

Assessment using the Edinburgh Handedness Inventory (Oldfield, 1971) led to the exclusion of 10 participants who were left-handed. The final sample comprised 32 participants (6 females and 26 males) with a mean age of 19.56 ± 0.84 years (body height: 1.71 ± 4.68 cm; body mass: 68.25 ± 7.64 kg). The participants' accumulated duration of engagement in badminton training was 2.5 ± 0.72 years. Furthermore, the mean laterality quotient for the 32 right-handed participants was 65 (ranging from 50 to 90).

Participants experienced an increase in somatic anxiety following the pressure induction, as evidenced by a significant increase in the mean score of the somatic anxiety scale, *t* (31) = −3.13, *p* = 0.004, *d* = −0.71. Additionally, the results indicated an increase in cognitive anxiety, *t* (31) = −1.33, *p* = 0.193, *d* = 0.24. These findings suggest that the pressure induction effectively elevated the participants' levels of somatic anxiety (Table 1). Cognitive anxiety was also elevated during the choking under pressure conditions, but this change was not statistically significant.

**Table 1:** Means (and standard deviations) of cognitive and somatic anxiety scores in choking under pressure conditions.

Conditions	Cognitive Anxiety		Somatic Anxiety	
	Pre	Post	Pre	Post
Choking Under Pressure	16.63 (4.44)	17.94 (7.34)	15.58 (3.64)	18.17 (5.65)

The results of the study demonstrate a significant improvement in badminton motor learning from pre- to post-tests after an eight-week intervention (Table 2). All participants showed an increase in choking under pressure effect during the execution of the three badminton skills, including the clear (*F* (1, 31) = 40.50, *p* = 0.001, partial η2 = 0.57), the serve (*F* (1, 31) = 29.82, *p* = 0.001, partial η2 = 0.49), and the wall volley (*F* (1, 31) = 49.03, *p* = 0.001, partial η2 = 0.61).

**Table 2:** Means (and standard deviations) of performance scores and differences.

Measures	Pressure-free		Post-pressure & Hemisphere-Specific Priming		
	Pre	Post	No Squeezing	Left-hand squeezing	Right-hand squeezing
Forehand Clear	23.66 (10.54)	40.19 (6.51)	30.84 (11.81)	32.19 (12.88)	35.47 (8.01)
Serve	34.09 (12.06)	62.22 (8.36)	44.53 (11.71)	46.06 (13.96)	43.81 (11.43)
Wall Volley	19.44 (6.52)	35.72 (5.56)	28. 88 (8.90)	32.41 (6.46)	31.09 (6.69)

In the high-pressure conditions, a significant benefit was found for right-hand squeezing during the forehand clear, compared to the no-squeezing condition (*p* = 0.04). However, the results of the TOST rejected the null hypothesis that right-hand squeezing and no-squeezing were different (*t* (31) = 2.31, *p* < 0.0139, *d* = 0.46), indicating that the two conditions were equivalent.

No significant differences were observed between the left- and right-hand squeezing and no-squeezing conditions during the serve (*p* = 1.00). The results of the TOST also rejected the null hypothesis that left- and right-hand squeezing and no-squeezing were different (left: *t* (31) = 3.71, *p* = 0.0004, *d* = 0.12; right: *t* (31) = 4.52, *p* = 0.0002, *d* = 0.06).

For the wall volley, a significant benefit was found for left-hand squeezing compared to the no-squeezing condition (*p* = 0.04). However, the results of the TOST rejected the null hypothesis that left- and right-hand squeezing and no-squeezing were different (left: *t* (31) = 2.55, *p* = 0.0079, *d* = 0.45; right: *t* (31) = 2.92, *p* = 0.0033, *d* = 0.28), indicating that the conditions were equivalent.

Pairwise comparisons were used to examine the effect of pressure on badminton skills performance. The results revealed a significant effect between the pressure-free conditions (conditions 1 & 2) and one post-pressure condition (condition 3: no-squeezing) across the three badminton skills. Specifically, the post-pressure (condition 3: no-squeezing) condition was found to be significantly less effective than the pressure-free (condition 2: post-test) condition in the forehand clear (*p* = 0.001). However, it was still higher than the pressure-free (condition 1: pre-test) condition (*p* = 0.0001). This trend was also observed in the serve and the wall volley.

## Discussion

### Pressure-Free Conditions

The current study aimed to investigate the potential moderating effect of increased hemispheric activity on motor skill failure under choking conditions in three badminton tasks, namely the forehand clear, the serve, and the wall volley. The study employed a pre-test/post-test experimental design to evaluate the impact of an 8-week badminton practice intervention on participants' performance in pressure-free conditions (condition 1 to condition 2) and under pressure conditions (conditions 3 to 5).

The results of the pressure-free conditions (conditions 1 & 2) indicated a significant improvement in participants' execution of the three badminton skills after the intervention. Specifically, the scores for all three skills almost doubled. The serve exhibited the most substantial improvement. These findings support prior research on badminton training and practice, which have consistently demonstrated improvements in skill execution and game performance (Hastie et al., 2022; Liu et al., 2020, 2021). For example, Liu et al. (2020) reported significant improvement in forehand clear skill levels among Physical Education major students from 29.58 to 46.47 points after a 15-week intervention, along with enhancements in tactical understanding and game performance. In the present study, participants were asked to perform three fundamental badminton skills without pressure, and the results showed that they developed solid and proficient motor skill patterns following the eight-week intervention. The observed improvements in the three fundamental badminton skills suggest that skills and precise execution can be attained and consolidated through structured practice in a pressure-free condition.

### Pressure-Induction Conditions

Participants in this study were placed under high levels of stress in order to examine the phenomenon of choking under pressure during the performance of three badminton skills in competition with their peers. Prior to their performance, somatic and cognitive anxiety were induced and measured. Somatic anxiety (Mesagno et al., 2019) is characterized by physiological symptoms such as an increased heart rate, rapid breathing, sweating, trembling, and muscular tension. It is typically triggered by various stressors, including physical exertion, social situations, or performance tasks. On the other hand, cognitive anxiety refers to the subjective experience of anxiety that involves worries, negative thoughts, and concerns about performance or outcomes (Gröpel and Mesagno, 2019).

Results of the study indicate that the purposefully created stressful environment, which included supportive audience, peers, skill competition, and possible rewards, significantly triggered participants' somatic anxiety. Verbal and nonverbal cues from the audience and peers likely contributed to increased heart rates, rapid breathing, sweating, and muscular tension, resulting in a choking effect under pressure (Gröpel and Mesagno, 2019; Mesagno et al., 2019).

While the increase in cognitive anxiety was not statistically significant, it is important to note that somatic and cognitive anxiety are distinct but interrelated experiences that can have significant implications for an individual's performance in tasks that require attention and concentration (Gröpel and Mesagno, 2019). The study highlights the importance of understanding the role of somatic and cognitive anxiety in performance under pressure. By inducing stress and measuring anxiety levels, researchers can gain insight into how these factors impact an individual's performance in various contexts.

Moving to the performance scores from pressure-free to post-pressure conditions, participants’ badminton skill execution significantly dropped from the no-squeezing condition (condition 3) in comparison to the pressure-free condition (condition 2). The decreased performance scores supported that players choked under pressure during their performance (Gorgulu and Gokcek, 2021). It is highly possible that their well-established automatic and unconscious motor patterns were disturbed by their somatic anxiety so that they were forced to pay attention to their movement and skill executions resulting in conscious processing (Cross-Villasana et al., 2015; Gallicchio et al., 2016; Hoskens et al., 2020).

The finding of a significant effect of pressure on badminton skills performance is consistent with previous studies (Masters and Maxwell, 2008; Wilson et al., 2009) that have demonstrated the negative impact of pressure on skilled motor performance. In the present study, the pressure-free conditions allowed participants to perform the badminton skills without any external pressure, resulting in improved motor skill execution (Liu et al., 2020). Conversely, in the post-pressure (no-squeezing) condition, participants were still able to execute the skills effectively but with reduced precision and accuracy, indicating the detrimental effects of pressure on skilled motor performance (Beckmann et al., 2012; Gröpel and Mesagno, 2019; Hoskens et al., 2020). Overall, the results of this study highlight the importance of managing pressure in sports performance to optimize motor skill execution.

Gröpel and Mesagno (2019) proposed various interventions to help athletes alleviate pressure, such as distraction, self-focus, and acclimatization. This study adopted a self-focus approach to see whether the choking effect could be reduced (Iwatsuki et al., 2018). The self-focus theory of choking under pressure suggests that increased conscious attention on the process of performance disrupts the automatic or overlearned nature of its execution, leading to motor pattern failure (Beckmann et al., 2021; Gallicchio et al., 2016; Masters and Maxwell, 2008). High-level athletes who experience choking under pressure have been found to have more interaction between the left temporal and frontal regions of the brain compared to athletes who do not experience choking (Gallicchio et al., 2016). This increased brain activation suggests that during the choking process, well-learned motor skills shift from being unconscious, automatic movements to conscious processing, which disrupts skilled performance (Mesagno et al., 2019).

Previous research has suggested possibilities for preventing the choking effect under pressure conditions by inhibiting the activity of the left hemisphere or enhancing the activity of the right hemisphere (Beilock and Gray, 2012). Researchers have also attempted to use priming to facilitate stimulus processing induced by prior exposure to a related stimulus. Left-hand contractions have been identified as one of the most effective techniques for combating choking in sport (Gröpel and Mesagno, 2019; Mesagno and Beckmann, 2017). Right-handed junior tennis players who performed a left-hand dynamic handgrip prior to serving were able to maintain service accuracy when under pressure (Gröpel and Mesagno, 2019).

This study added hand-squeezing as a priming strategy before performing badminton skills in a highly stressed condition. However, the results did not show any motor skill execution difference between no-squeezing, left-hand squeezing, and right-hand squeezing across all three badminton skills. These results are in line with Hoskens et al.’s (2020) outcomes with novice golfers who did not find a positive effect of left-hand squeezing on golf putting performance. These findings, however, were contradictory to previous studies that reported unilateral hand contractions functioning as a priming strategy to prevent reduced accuracy when players choked under pressure (Beilock and Gray, 2012; Hoskens et al., 2020).

Although Beilock and Gray (2012) reported positive results regarding ball-squeezing to prevent the choking effect in the badminton serve test, the ball-squeezing task did not allow for precise regional specification of hemispheric activation as expected. Players in the present study improved their serve scores from 34.09 to 62.22 after eight weeks of intervention in a pressure-free condition. However, their serve scores significantly dropped from 62.22 to less than 50 points when pressure was induced during their performance. Although left-hand squeezing showed higher scores in the serve and the wall volley, the one-sided *t*-tests (TOST) did not support the difference between left- and right- hand squeezing.

One possible reason for the difference in serve scores between the present study and Beilock and Gray's (2012) study is that the pressure induction procedure was different. In Beilock and Gray's study, only competition and reward were introduced to help elicit pressure in participants. In contrast, in this study, there were 100 supporting audiences randomly split into two teams to play against each other, as well as rewards and competitions. This highly stressful climate mixed with verbal and nonverbal cues from supporting audiences, peers, competition pressures, and worry about performance may have prevented players from deactivating right hemisphere activities with the 30-s left-hand ball squeezing.

Handedness and game experience are other potential explanations for the phenomenon of choking in athletes. Gröpel and Mesagno (2019) found that right-handed athletes were more likely to experience choking than their left-handed counterparts in a sporting context. The present study excluded left-handed players to reduce confounding factors and detect the priming intervention effect. However, this also prevents confirmation of potential behavioral differences between left- and right-handed participants during priming strategies.

High-level athletes who experience choking under pressure have been found to have increased interaction between the left temporal and frontal regions of the brain compared to athletes who do not experience choking (Cross-Villasana et al., 2015; Gallicchio et al., 2016). This increased brain activation suggests that during the choking process, well-learned motor skills shift from being unconscious, automatic movements to conscious processing, which disrupts skilled performance (Beckmann et al., 2012, 2013; Gallicchio et al., 2016).

It is important to note that participants in this study were not elite athletes who had abundant experience with various games to competently handle pressure by adopting various strategies such as distraction, self-focus, and acclimatization (Gallicchio et al., 2016). They only had two years of badminton training and limited exposure to high-stressful environments for performing badminton skills. As a result, participants failed to alleviate their pressures with priming after heavily choking because they lacked experience in dealing with choking under pressure conditions (Jooste et al., 2023).

Further research is needed to explore potential differences in choking behavior between left- and right-handed athletes and to investigate the efficacy of priming strategies in elite athletes with significant game experience (Beckmann et al., 2012, 2013). By doing so, coaches and athletes may be able to better understand and address the complex factors contributing to choking and develop effective interventions to mitigate its impact on performance.

Some methodological constraints may have impeded the outcomes of this study. One limitation pertains to the absence of precise knowledge regarding the neurophysiological discrepancies between left- and right-handed athletes while executing badminton tasks. Although the study design drew from EEG observations associated with choking and handedness literature (Gallicchio et al., 2016), the absence of EEG instrumentation precludes identification of specific patterns of brain activation that may have led to choking among the athletes. Consequently, future investigations may incorporate EEG analyses to delineate the cortical activation differences between right- and left-handed athletes when performing a specific sport task under pressure with priming.

Another shortcoming of this study concerns the sample size, which was restricted to right-handed participants. Excluding left-handed individuals from the sample led to a smaller sample size and a within-subject design. While group numbers aligned with those found in numerous prior studies on choking (Beckmann et al., 2013) and handedness (Serrien et al., 2012), replicating the current study with a larger number of participants and adopting a within-and-between subject design could increase the study's power and yield more conclusive results.

## Conclusions

The study provides evidence for the assumption that participants’ motor skills execution significantly improved after 8 weeks of intervention and their performance decrements evoked by the high-pressure condition. Moreover, the priming strategy (hand squeezing) did not seem to alleviate the pressures to benefit their performance.

## References

[ref1] Beckmann, J., Fimpel, L., and Wergin, V.V. (2021). Preventing a loss of accuracy of the tennis serve under pressure. PLoS ONE, 16, e0255060. 10.1371/journal.pone.0255060 34310638 PMC8312934

[ref2] Beckmann, J., Gröpel, P., and Ehrlenspiel, F. (2012). Preventing motor skill failure through hemisphere-specific priming: Cases from choking under pressure. Journal of Experimental Psychology: General, 141, 764–778. 10.1037/a0029852 22946898

[ref3] Beckmann, J., Gröpel, P., and Ehrenspiel, F. (2013). Preventing motor skill failure through hemisphere-specific priming: Cases from choking under pressure. Journal of Experimental Psychology: General, 142, 679–691. 10.1037/a0029852 22946898

[ref4] Beilock, S. L., and Gray, R. (2012). From attentional control to attentional spillover: A skill-level investigation of attention, movement, and performance outcomes. Human Movement Science, 31, 1473–1499. 10.1016/j.humov.2012.02.014 23182433

[ref5] Cox, R. H., Martens, M. P., and Russell, W. D. (2003). Measuring anxiety in athletics: The Revised Competitive State Anxiety Inventory-2. Journal of Sport and Exercise Psychology, 25, 519–533. 10.1123/jsep.25.4.519

[ref6] Cross-Villasana, F., Gröpel, P., Doppelmayr, M., and Beckmann, J. (2015). Unilateral left-hand contractions produce widespread depression of cortical activity after their execution. PLoS ONE, 10, e0145867. 10.1371/journal.pone.0145867 26709832 PMC4692494

[ref7] Gallicchio, G., Cooke, A., and Ring, C. (2016). Lower left temporal-frontal connectivity characterizes expert and accurate performance: High-alpha T7-Fz connectivity as a marker of conscious processing during movement. Sport, Exercise, and Performance Psychology, 5, 14. 10.1037/spy0000055

[ref8] Gorgulu, R., & Gokcek, E. (2021). The Effects of Avoiding Instructions Under Pressure: An Examination of the Volleyball Serving Task. Journal of Human Kinetics, 78, 239–249. 10.2478/hukin-2021-0039 34025881 PMC8120970

[ref9] Gröpel, P., and Mesagno, C. (2019). Choking interventions in sports: A systematic review. International Review of Sport and Exercise Psychology, 12, 176–201. 10.1080/1750984X.2017.1408134

[ref10] Hastie, P. A., Wang, W., Liu, H., and He, Y. (2022). The effects of play practice instruction on the badminton content knowledge of a cohort of Chinese physical education majors. Journal of Teaching in Physical Education, 41, 347–355. 10.1123/jtpe.2021-0075

[ref11] Hoskens, M. C. J., Bellomo, E., Uiga, L., Cooke, A., and Masters, R. S. W. (2020). The effect of unilateral hand contractions on psychophysiological activity during motor performance: Evidence for verbal-analytical engagement. Psychology of Sport and Exercise, 48, 101668. 10.1016/j.psychsport.2020.101668

[ref12] Iwatsuki, T., Raalte, J. L. V., Brewer, B. W., Petitpas, A., & Takahashi, M. (2018). Relations Among Reinvestment, Self-Regulation, and Perception of Choking Under Pressure. Journal of Human Kinetics, 65, 281–290. 10.2478/hukin-2018-0042 30687439 PMC6341961

[ref13] Jooste, J., Kruger, A., & Tinkler, N. (2023). The Influence of Emotional Intelligence on Coping Ability in Senior Female Field-Hockey Players in South Africa. Journal of Human Kinetics, 87, 211–223. 10.5114/jhk/161550 37229407 PMC10203831

[ref14] Kirkpatrick, N. J., Ravichandran, V. J., Perreault, E. J., Schaefer, S. Y., and Honeycutt, C. F. (2018). Evidence for startle as a measurable behavioral indicator of motor learning. PLoS ONE, 13, e0195689. 10.1371/journal.pone.0195689 29742130 PMC5942773

[ref15] Liao, C., and Masters, R. S. (2002). Self-focused attention and performance failure under psychological stress. Journal of Sport and Exercise Psychology, 24, 289–305. 10.1123/jsep.24.3.289 28682203

[ref16] Liu, H. R., Wang, W., He, Y. H., and Hastie, P. (2020). The impact of play practice on Chinese physical education pre-service teachers badminton content knowledge. Asian Journal of Kinesiology, 22, 17–23. 10.15758/ajk.2020.22.3.17

[ref17] Liu, H. R., Wang, W., Zhang, C. H., and Hastie, P. A. (2021). College students’ development of badminton skills and tactical competencies following play practice. Journal of Teaching in Physical Education, 40, 284–292. 10.1123/jtpe.2019-0292

[ref18] Masters, R., and Maxwell, J. (2008). The theory of reinvestment. International Review of Sport and Exercise Psychology, 1, 160–183. 10.1080/17509840802287218

[ref19] McKeone, L. (2023). Belgium’s Romelu Lukaku punches wall, breaks down crying after choking vs. Croatia. Available online: https://www.thebiglead.com/posts/romelu-lukaku-belgium-world-cup-choke-croatia-punch-crying-video-01gk7a04fvdt (accessed on 1 February 2023).

[ref20] Mesagno, C., and Beckmann, J. (2017). Choking under pressure: Theoretical models and interventions. Current Opinion in Psychology, 16, 170–175. 10.1016/j.copsyc.2017.05.015 28813345

[ref21] Mesagno, C., Garvey, J., Tibbert, S. J., and Gröpel, P. (2019). An investigation into handedness and choking under pressure in sport. Research Quarterly for Exercise and Sport, 90, 217–226. 10.1080/02701367.2019.1588935 30920352

[ref22] Oldfield, C. (1971). The assessment and analysis of handedness: the Edinburgh inventory. Neuropsychologia, 9, 97–113. 10.1016/0028-3932(71)90067-4 5146491

[ref23] Rink, J. E., French, K. E., and Greham, K. C. (1996). Implication for practice and research. Journal of Teaching in Physical Education, 15, 490–502. 10.1123/jtpe.15.4.490

[ref24] Serrien, D. J., Sovijärvi-Spapé, M. M., and Farnsworth, B. (2012). Bimanual control processes and the role of handedness. Neuropsychology, 26, 802–807. 10.1037/a0030154 23106119

[ref25] Wilson, M. R., Vine, S. J., and Wood, G. (2009). The influence of anxiety on visual attentional control in basketball free throw shooting. Journal of Sport and Exercise Psychology, 31, 152–168. 10.1123/jsep.31.2.152 19454769

